# The El Niño Southern Oscillation drives multidirectional inter-reef larval connectivity in the Great Barrier Reef

**DOI:** 10.1038/s41598-022-25629-w

**Published:** 2022-12-09

**Authors:** Rodrigo Gurdek-Bas, Jessica A. Benthuysen, Hugo B. Harrison, Kyall R. Zenger, Lynne van Herwerden

**Affiliations:** 1 AIMS@JCU, Division of Research & Innovation, James Cook University and Australian Institute of Marine Science, Townsville, 4811 Australia; 2 College of Science and Engineering, James Cook University, Townsville, 4811 Australia; 3 Australian Institute of Marine Science, Townsville, 4810 Australia; 4 ARC Centre of Excellence for Coral Reef Studies, James Cook University, Townsville, 4811 Australia

**Keywords:** Marine biology, Physical oceanography

## Abstract

The El Niño Southern Oscillation (ENSO) is the strongest source of interannual global climate variability, and extreme ENSO events are projected to increase in frequency under climate change. Interannual variability in the Coral Sea circulation has been associated with ENSO, although uncertainty remains regarding ENSO's influence on hydrodynamics and larval dispersal in the adjacent Great Barrier Reef (GBR). We investigated larval connectivity during ENSO events from 2010 to 2017 throughout the GBR, based on biophysical modelling of a widespread predatory reef fish, *Lutjanus carponotatus*. Our results indicate a well-connected system over the study period with high interannual variability in inter-reef connectivity associated with ENSO. Larval connectivity patterns were highly correlated to variations in the Southern Oscillation Index (SOI). During El Niño conditions and periods of weak SOI, larval dispersal patterns were predominantly poleward in the central and southern regions, reversing to a predominant equatorward flow during very strong SOI and extreme La Niña conditions. These ENSO-linked connectivity patterns were associated with positive connectivity anomalies among reefs. Our findings identify ENSO as an important source of variation in larval dispersal and connectivity patterns in the GBR, which can influence the stability of population dynamics and patterns of biodiversity in the region.

## Introduction

The El Niño Southern Oscillation (ENSO) is one of Earth’s most important climatic phenomena and a source of year-to-year global climate variability^1^. Generated in the equatorial Pacific Ocean, ENSO alternates between El Niño and La Niña phases, modifying the interannual atmospheric and ocean circulation in this region^2^. During El Niño (or La Niña), anomalously warm (or cool) sea surface temperatures are found in the central and eastern Pacific Ocean, inducing weakened (or strengthened) easterly winds^1,2^. Climate projections indicate extreme El Niño and La Niña events will increase in frequency due to increasing sea surface temperatures associated with climate change^2–4^. Such events already negatively impact marine ecosystems^5–7^, however, the role of ENSO, including ENSO extremes, in regulating seascape connectivity in the ocean requires further research.

**Figure 1:**
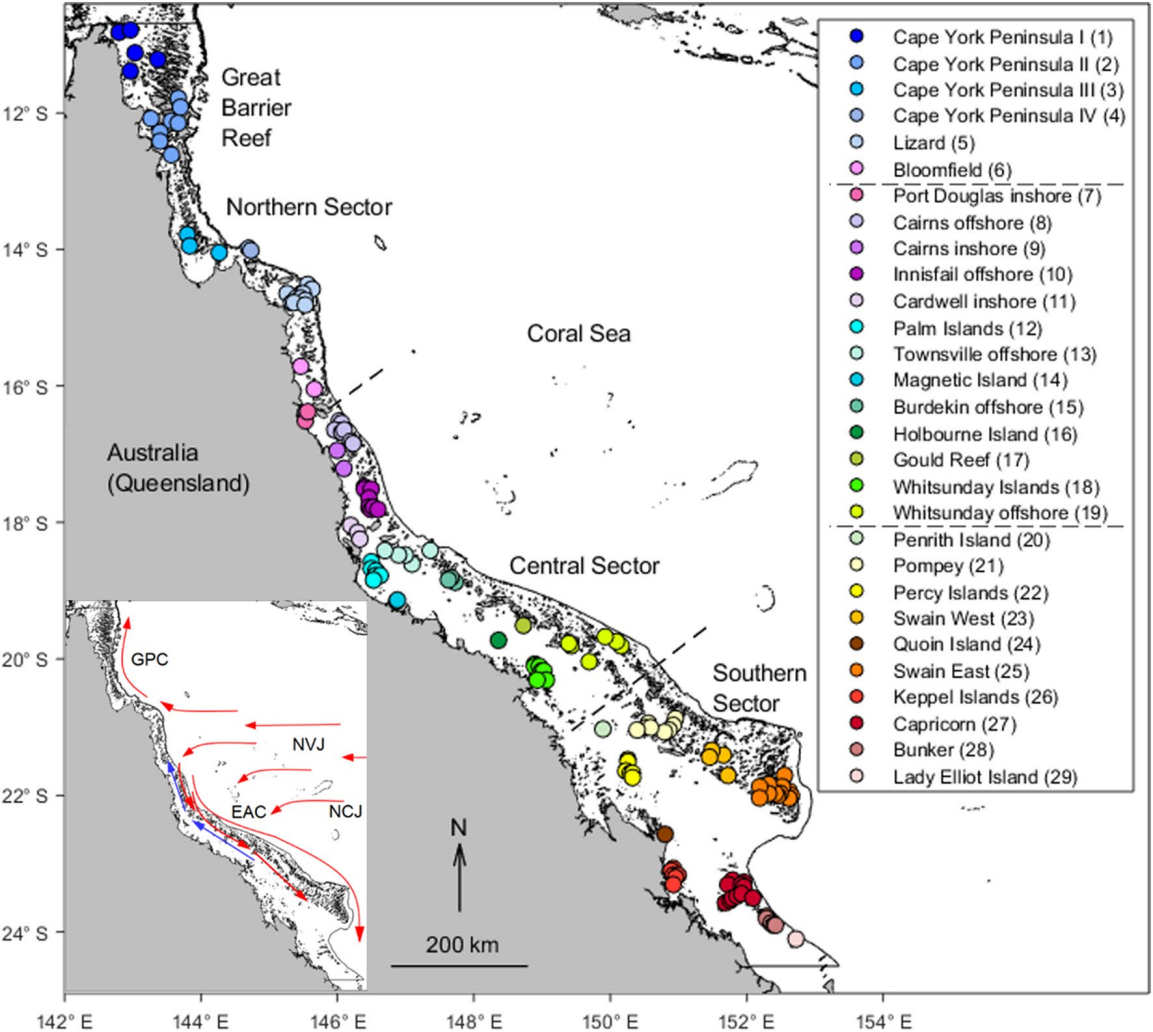
Regions of larval sources and destinations of modelled larval connectivity in the GBR, based on *L. carponotatus* occurrence records. Each region is indicated by a colour (see upper inset colour key) and consists of a grouping of local populations. Region names in the legend are ordered from north to south (meridional axis), and the same order is used for representation of regions in the connectivity matrices. Northern, central and southern GBR sectors are divided by dashed black lines. The black line offshore (following the length of the coast) delimits the GBR shelf and corresponds to the 100 m isobath. Lower inset: The South Equatorial Current (SEC) is composed of two Coral Sea branches, the North Vanuatu Jet (NVJ) and the North Caledonian Jet (NCJ). The NVJ branch crosses the Coral Sea westwards, reaching the outer GBR and bifurcating equatorward, forming the Gulf of Papua Current (GPC), and poleward, forming the EAC, both of which flow adjacent to the east Australian continental shelf edge^47^. Predominant and transient surface currents during the main *L*. *carponotatus* spawning season are shown by red and blue arrows, respectively. In the GBR, red arrows indicate the oceanic inflow from the Coral Sea and predominant poleward flow, and blue arrows show the reversal of the dominant flow, exhibited during ENSO conditions. The figure was created using MATLAB v9.4, available at https://www.mathworks.com/products/matlab.html.

ENSO dominates interannual transport variability in the Coral Sea, with the Southern Oscillation Index (SOI), an index that gauges ENSO’s phase and strength, well-correlated with the South Equatorial Current (SEC) transport^8^. Along the Great Barrier Reef (GBR) shelf adjacent to the Coral Sea, ocean circulation is driven in part by cross-shelf pressure gradients associated with the East Australian Current (EAC) and Coral Sea intrusions^9–11^, with interannual fluctuations possibly modulated by ENSO^11,12^ (see Fig. 1—lower inset—for details on the ocean currents in the Coral Sea and GBR). Long-term along-shelf current predictions in the central GBR have identified strong southward flows coinciding with El Niño events^13^. Changes in ENSO activity can also be related to interannual fluctuations in rainfall and river flow discharge, with higher flows to the GBR associated with La Niña events^14^. These river plumes often move equatorward along the coast^15^, particularly during very strong La Niña events^16^.

Variation in large-scale climatic conditions (SOI) has also been strongly correlated to reef fish recruitment^17^. Furthermore, a strong relationship has been found between the timing of ENSO, including extreme events, and coral reef fish larval supply^18^. Biophysical modelling of fish larval dispersal has suggested that changes in ENSO patterns affect dispersal trajectories and distances^19^. Importantly, interannual variation in the scale and consistency of coral reef fish larval dispersal patterns has important implications for metapopulation persistence^20^. Given the influence of ENSO on ocean circulation, and the importance of marine connectivity on the persistence of populations^21^, quantifying connectivity patterns throughout marine seascapes under different ENSO scenarios constitutes a relevant issue.

Coral reef ecosystems such as the GBR are naturally patchy, and the degree of connectivity among reefs is an important determinant of fish recruitment, metapopulation dynamics and genetic exchange over regional scales^22–24^. Biophysical modelling of reef fish larval connectivity in the northern GBR suggests a highly connected system^25^, although temporal changes in hydrodynamics can lead to interannual inter-reef connectivity fluctuations^22^. Furthermore, dispersal during ENSO events may influence coral reef fish population synchrony in the GBR, with synchronous fluctuations correlated with ENSO^26^. Although there is some evidence of ENSO’s impacts on reef fish larval supply, the influence ENSO has on larval connectivity patterns across the regional scales and complexity of large coral reef ecosystems, such as the GBR, needs further study.

In this study, we quantify larval connectivity patterns throughout the GBR from 2010 to 2017, based on biophysical modelling of stripey snapper, *Lutjanus carponotatus* (Lutjanidae) larval dispersal. The study period spans four ENSO events, including one of the strongest La Niña events on record (2010–2011), a moderate La Niña (2011–2012), an El Niño alert (2014–2015) and a strong El Niño (2015–2016), as well as intermittent neutral states (Table 1). Specifically, our objectives were to (i) estimate the degree of larval connectivity among reefs over time and examine the temporal variation in annual connectivity, (ii) explore the relationship between interannual larval connectivity patterns and different ENSO conditions, and (iii) identify larval connectivity anomalies during El Niño and La Niña events. We hypothesise that interannual connectivity patterns in the GBR vary due to changes in ENSO conditions, with either (1) predominant poleward larval connectivity for the central to southern GBR during El Niño events or (2) predominant equatorward larval connectivity during extreme La Niña events.

**Table 1:** The SOI during modelled larval dispersal periods in the GBR.

Year	October	November	December	January (next year)	Main ENSO phase
2010	18.3	16.4	27.1	19.9	Very strong La Niña
2011	7.3	13.8	23	9.4	Moderate La Niña
2012	2.4	3.9	− 6	− 1.1	Neutral
2013	− 1.9	9.2	0.6	12.2	Neutral
2014	− 8	− 10	− 5.5	− 7.8	El Niño alert
2015	− 20.2	− 5.3	− 9.1	− 19.7	Strong El Niño
2016	− 4.3	− 0.7	2.6	1.3	Neutral–La Niña
2017	9.1	11.8	− 1.4	8.9	Neutral–La Niña

Data obtained from the Australian Bureau of Meteorology (http://www.bom.gov.au/climate/enso). The main ENSO phases during the 8-year study period are indicated annually.

## Materials and methods

### ENSO events

We used the SOI to identify the ENSO phases for each modelled larval dispersal event, between October and January (austral spring–early summer) from 2010 to 2017 inclusive (Table 1). Larval dispersal events were selected to coincide with the spawning peak of *L. carponotatus* in the GBR^27^. The SOI indicates the development and strength of El Niño or La Niña events in the Pacific Ocean, by measuring surface air pressure differences between Tahiti and Darwin (Australia) as an indicator of the intensity of the Walker Circulation^28^. We used the SOI provided by the Australian Bureau of Meteorology (http://www.bom.gov.au/climate/enso). The method the Bureau of Meteorology uses to calculate the SOI is based on mean and standard deviation of the pressure differences between Tahiti and Darwin, with the climatology period based on a dataset from 1933 to 1992^29^. Sustained negative (below − 7) or positive (above + 7) SOI values typically indicate El Niño or La Niña events, respectively. The following ENSO events were identified within the study time frame: very strong 2010 La Niña (2010–2011), moderate 2011 La Niña (2011–2012), 2014 El Niño alert (2014–2015) and strong 2015 El Niño (2015–2016)^28^ (http://www.bom.gov.au/climate/enso) (Table 1). ENSO events tend to decay by austral autumn of the following year^28^. During 2012 (2012–2013) and 2013 (2013–2014) neutral ENSO states prevailed, while 2016 (2016–2017) and 2017 (2017–2018) exhibited a mix of neutral and La Niña conditions (Table 1).

### Hydrodynamic model

The eReefs project (http://ereefs.org.au) includes a hydrodynamic model that spans the whole GBR^30^. The eReefs modelling system includes a 4 km and 1 km resolution model domain providing near-real time data since September 2010 and December 2014, respectively. The eReefs hydrodynamic model is based on the Sparse Hydrodynamic Ocean Code (SHOC), which is a finite difference, three-dimensional model based on equations of momentum, continuity and conservation of heat and salt, which uses Boussinesq and hydrostatic approximations, discretised on an Arakawa C grid^31^. The eReefs hydrodynamic model is forced by wind, surface heat and water fluxes provided by the Bureau of Meteorology’s Australian Community Climate and Earth System Simulation (ACCESS-R; 12 km resolution). The regional model is forced along the boundaries by low frequency ocean currents from the Bureau of Meteorology’s Ocean Modelling Analysis and Prediction System (OceanMAPS), which is a global ocean model^32^. The tidal component is implemented from the global CSR tide model^33,34^. Freshwater inputs to the GBR representing the major rivers are obtained from the Department of Natural Resources, Mines and Energy gauging network^30^. Model outputs include three-dimensional distributions of velocity, temperature, salinity, density, passive tracers, mixing coefficients and sea-level. The hydrodynamic model has been validated at key locations along the GBR (from Lizard Island in the northern GBR to the Capricorn-Bunker Group in the southern GBR) and is a good indicator of currents in those regions^30,35^.

### Larval dispersal and connectivity modelling

GBR-wide larval connectivity was estimated using Connie3, a high-resolution advection/diffusion model of the whole GBR^36^ (https://connie.csiro.au/). Connie3 was used with the eReefs hydrodynamic model velocity data at 4 km resolution scale (to include the climatic events since 2010) and a temporal resolution of one hour. A fourth-order Runge–Kutta ordinary differential equation solver subsequently tracked individual particles using the horizontal velocity at the specified depth and time. Connie has been successfully used to model larval transport in the ocean, including fish larval dispersal off northwestern Australia^37^ and along the Queensland coast (including the GBR) and the Coral Sea^38^. Connie has also been used to model connectivity networks of crown-of-thorns starfish^39^ and corals^40^ in the GBR.

Larval connectivity was investigated among 29 regions distributed along and across the GBR, according to *L. carponotatus* occurrence records^41^ (Fig. 1). Regions referred to as “offshore” included mid- to outer-shelf reefs. *Lutjanus carponotatus* is a widely distributed, dominant predatory fish across the GBR^42^. *Lutjanus carponotatus* has been used as a model species to investigate larval connectivity along the coast of northwestern Australia because of its similar larval settlement behaviour and ecology to other predatory fishes^43^. The average pelagic larval duration of *L. carponotatus* in the GBR is reported as 25 days^44^. Empirical studies of *L. carponotatus* larval dispersal in the GBR have confirmed dispersal up to ~ 30 km^45^. Furthermore, larvae likely disperse much further as population genetic work on adult population structure along the central and southern GBR identified no population subdivision, suggesting that island groups are connected by larval dispersal^46^.

Modelled larvae were seeded between October and December from 2010 and 2017 from 141 locations in the GBR (Fig. 1). Larvae were released at a constant rate of 100 particles/grid cell/day to capture all possible spawning events and allowed to disperse over 25 days. *Lutjanus carponotatus* larval vertical distribution was informed by larval development and behavioural data along with data from other lutjanids^48–50^. Larval depth preferences were set to correspond with the depth distribution layers in Connie3 as follows: day 1 at 1 m, days 2 to 20 at 3 m, and days 21 to 25 at 6 m depths. Dispersing larvae were subjected to an 18% daily mortality rate, as proposed for marine pelagic larval mortality rates^22^. Hypothetical sensory zones of 4 km surrounding reef habitats were created based on larval fish sensory capabilities^51^, and the applied model resolution scale. The buffer zone was created using a Geographic Information System, QGIS 2.18.0 (QGIS Development Team 2018, QGIS Association, https://www.qgis.org) (and geoprocessing tools within QGIS). Larvae that reached the reef sensory zones by the end of their pelagic larval duration were considered able to settle. Larvae were subjected to 13% mortality due to predation while attempting to settle as suggested for *L. carponotatus*^49^. After the pelagic larval stage, larvae that did not reach the sensory zones were excluded from subsequent analyses.

### Quantifying spatial and temporal connectivity patterns

Annual connectivity values were determined among 29 GBR regions from 2010 to 2017, and eight GBR-wide annual connectivity matrices were created (see Supplementary Fig. S1). Connectivity matrices show the probability that larvae spawned at a given origin (out of 29 regions) disperse and recruit to a given destination (out of 29 potential regions). Larval retention was defined as the fraction of larvae produced at a given region that settled into that region^21^.

Larval connectivity was explored overall (averaged) from 2010 to 2017 to estimate the degree of larval connectivity among regions over the study period. Larval connectivity was also explored in each year to identify annual variation in connectivity patterns. The coefficient of variation (CV) of yearly larval connectivity (from 2010 to 2017) between each pair of regions was calculated as a measure of temporal variation in connectivity, according to: CV = (connectivity standard deviation/mean connectivity). In addition, the coefficient of determination (r^2^) between averaged larval connectivity among regions (from 2010 to 2017) and CV of among region connectivity was explored to assess stability in the levels of the connectivity.

The relationship between interannual larval connectivity patterns in the GBR and different ENSO conditions (i.e. variations in the SOI) was assessed over the study period by the coefficient of determination (r^2^). The SOI was averaged yearly (during 2010 to 2017) from values in Table 1. Equatorward, poleward and across-shelf larval connectivity was measured during each annual spawning season from each region and averaged throughout the central and southern GBR. Equatorward and poleward connectivity refers to northward and southward larval connectivity, respectively, along the continental shelf (i.e. among inshore or offshore regions). Across-shelf connectivity includes larval connectivity from offshore towards inshore regions. The central and southern GBR sector was selected to explore this relationship because the greatest changes in larval dispersal patterns occurred there. The southernmost GBR regions (including the Capricorn-Bunker–Lady Elliot regions and the adjacent inner islands) were excluded from this relationship because dispersal patterns in this area were relatively different in terms of directionality. A significance level of 0.05 was considered in all relationships.

Positive and negative anomalies in larval connectivity among regions were calculated yearly to identify connectivity anomalies during ENSO events. Eight GBR-wide annual connectivity anomaly matrices were generated, including the 2010 and 2011 La Niña, and 2014 and 2015 El Niño events (see Supplementary Fig. S2). For each annual matrix, the larval connectivity patterns for that year are represented, and it is indicated whether connections showed a positive or negative anomaly (i.e. an increase or a decrease, respectively) in connectivity relative to the 2010–2017 mean (connectivity).

### Ocean, riverine discharge and wind circulation patterns

Hydrodynamic data was provided through the eReefs project. Near-surface ocean currents and salinity (as a proxy of river discharge) from the 4-km horizontal resolution eReefs hydrodynamic model (GBR4 v2), and wind data from ACCESS-R, were obtained for the larval dispersal periods. Ocean current velocity (m s^−1^) and salinity values were obtained at a depth of − 1.5 m, and wind velocity (m s^−1^) values were obtained at 10 m height. Ocean current, wind velocity and salinity values were averaged over time (between October and January) for each of the years examined (2010 to 2017). Ocean, riverine discharge and wind circulation patterns are described in Supplementary information (see Supplementary Results and Figs. S4–S6).

## Results

### GBR-wide larval connectivity and temporal variation

**Figure 2:**
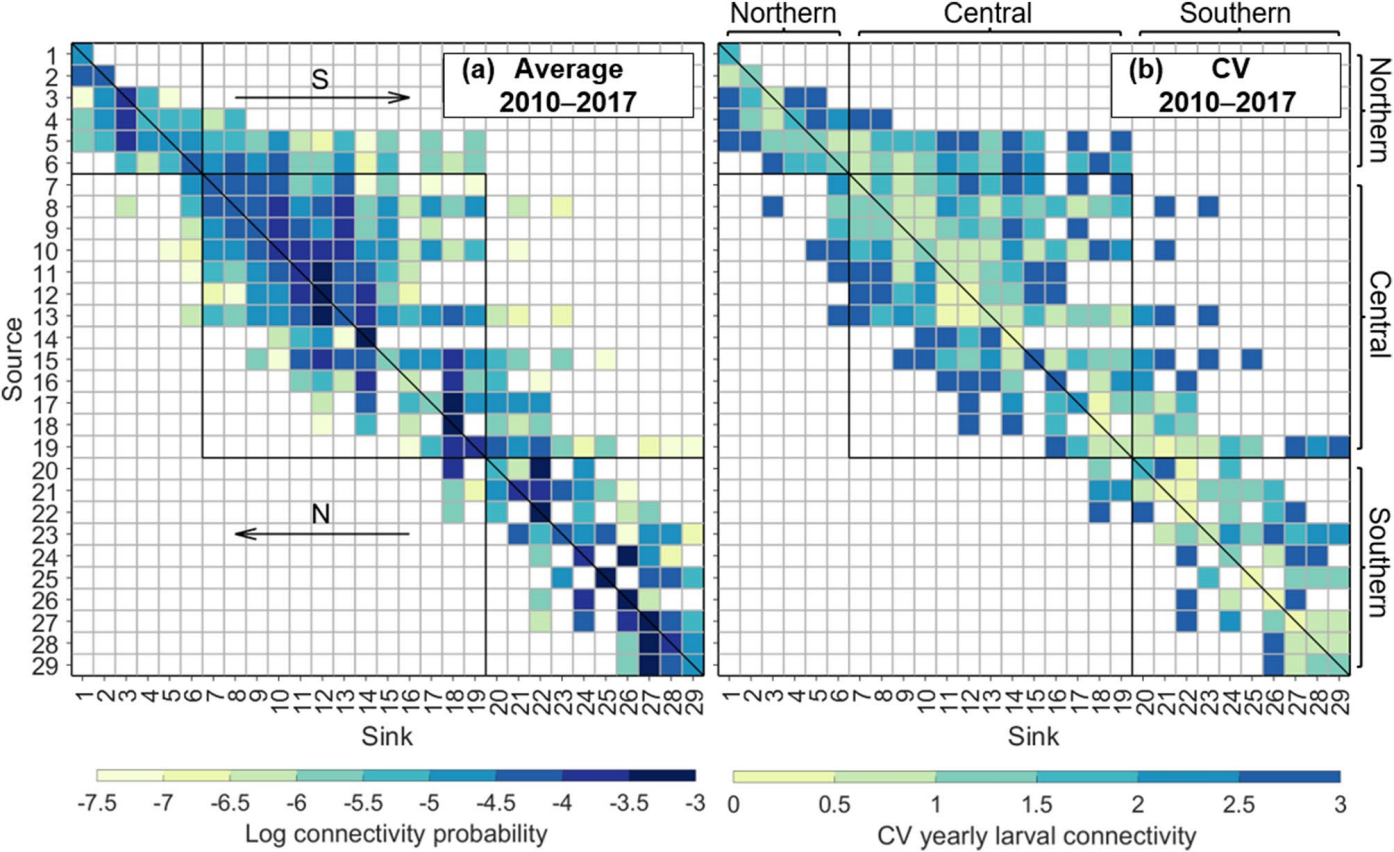
Connectivity patterns for modelled *L. carponotatus* larvae in the GBR. Particles were released annually from 2010 to 2017 (between October and December) from source regions (rows) and recruited to sink regions (columns). (**a**) Connectivity matrix representing the average annual larval connectivity among regions. Values along the diagonal represent larvae settled into the same region. (**b**) Coefficient of variation (CV) matrix of annual larval connectivity. Values along the diagonal represent CV of larval retention. In both plots, values to the right or left of the diagonal indicate poleward (“S” for southward–southeastward) or equatorward (“N” for northward–northwestward) connectivity. The northern, central and southern GBR sectors are bordered by black lined squares. Regions are ordered from north to south following the same order as presented in the legend of the upper inset in Fig. 1. The figure was created using MATLAB v9.4, available at https://www.mathworks.com/products/matlab.html.

Average connectivity patterns in the GBR revealed large-scale, multidirectional larval connectivity among reefs throughout the system, with bi-directional larval dispersal along the latitudinal and across-shelf gradients (Fig. 2a). Larval connectivity in the central and southern GBR was predominantly poleward (61% of connections) and resulted in longer-distance dispersal events towards southern reefs than equatorward dispersal events (e.g. rows 7–10, 19, 23; Fig. 2a). Larvae sourced from the far northern GBR (< 14°S) dispersed mostly equatorward (e.g. rows 2–3; Fig. 2a). Larval retention was common in all GBR regions (values along the diagonal), although it was variable between regions (Fig. 2a) and over time (Fig. 2b).

The degree of larval connectivity among reefs throughout the GBR was highly variable, both spatially and temporally (Fig. 2b). The CV in connectivity values ranged from 0.32 to 2.83, with a median distribution of 1.84 (Fig. 2b), indicating very large temporal fluctuations in connectivity between years. Higher CV values were identified in equatorward dispersal events and over longer-distances poleward (Fig. 2b). The strength of connectivity significantly decreased with higher CV (r^2^ = 0.57, P < 0.00001, exponential decay; Fig. 2; see Supplementary Fig. S3), suggesting that the strongest connectivity patterns were more consistent in time, but weaker and longer dispersal events were more variable.

### ENSO-linked connectivity patterns

**Figure 3:**
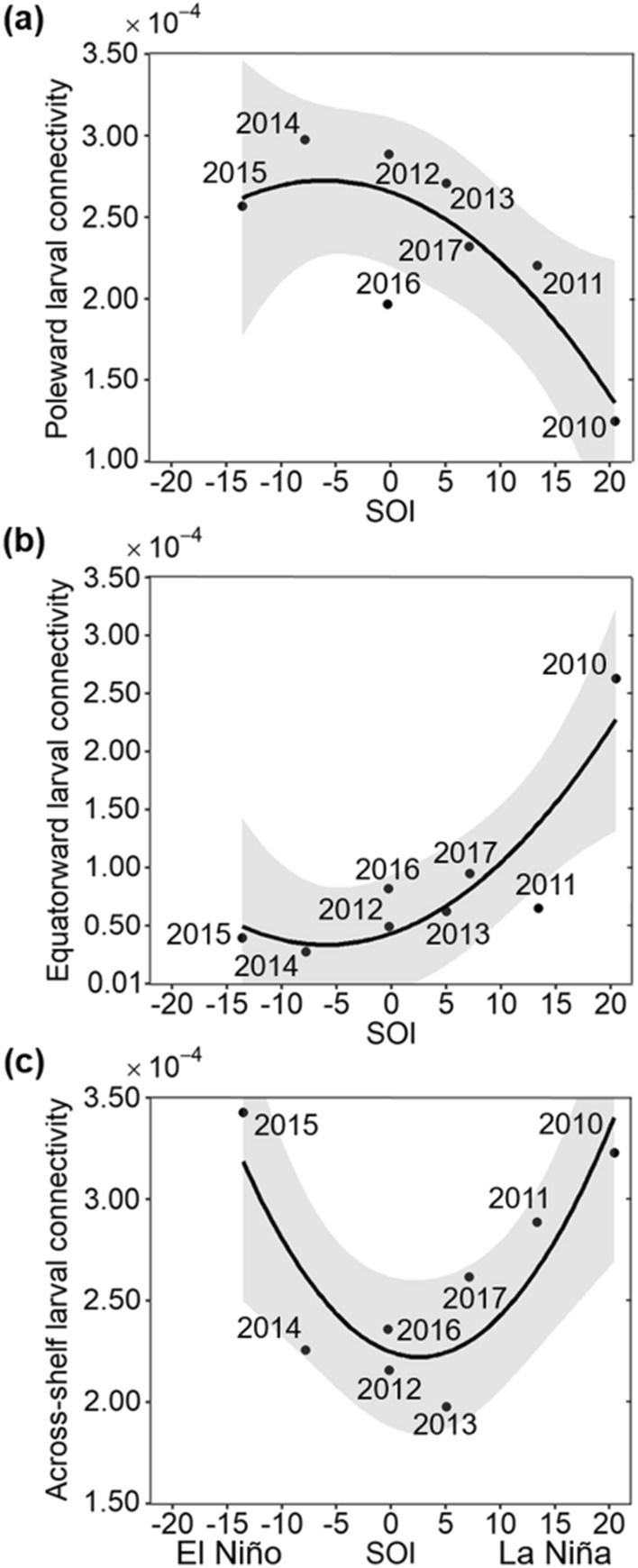
Relationship between interannual connectivity patterns for modelled *L. carponotatus* larvae in the central and southern GBR and the Southern Oscillation Index (SOI). Connectivity patterns are represented according to (**a**) poleward, (**b**) equatorward and (**c**) across-shelf connectivity probability values. Variables were averaged for each year from 2010 to 2017. The following ENSO events were identified: very strong 2010 La Niña, moderate 2011 La Niña, 2014 El Niño alert and strong 2015 El Niño (during 2012, 2013, 2016 and 2017 a mix of neutral and La Niña conditions prevailed). Grey shaded areas show 95% confidence intervals of the connectivity means. All relationships (r^2^) were statistically significant at a P < 0.05.

The relationship between interannual larval connectivity patterns in the (central and southern) GBR and different ENSO conditions was explored for an SOI range between -14 (i.e. the strong 2015 El Niño) and 20 (i.e. the very strong 2010 La Niña) (Fig. 3). Mean poleward connectivity significantly decreased with an increase of the SOI (r^2^ = 0.68, P < 0.05; Fig. 3a).This relationship indicated that greater poleward connectivity patterns occurred during El Niño events and weak SOI (Fig. 3a). In contrast, lower poleward connectivity was exhibited during the extreme 2010 La Niña event (Fig. 3a). Conversely, mean equatorward connectivity significantly increased with an increase of the SOI (r^2^ = 0.78, P < 0.05; Fig. 3b). Accordingly, stronger equatorward connectivity patterns occurred during the extreme 2010 La Niña conditions, but weaker equatorward connectivity was exhibited during El Niño events (Fig. 3b). Over the study period, poleward connectivity was greater than equatorward connectivity, except during the 2010 La Niña event (Fig. 3a,b). A significant relationship was found between mean across-shelf connectivity and the SOI (r^2^ = 0.76, P < 0.05; Fig. 3c). This relationship showed an increase in across-shelf connectivity at both lowest and highest SOI values, but a decrease at intermediate SOI values (Fig. 3c).

### Larval connectivity during the 2014 and 2015 El Niño events

**Figure 4:**
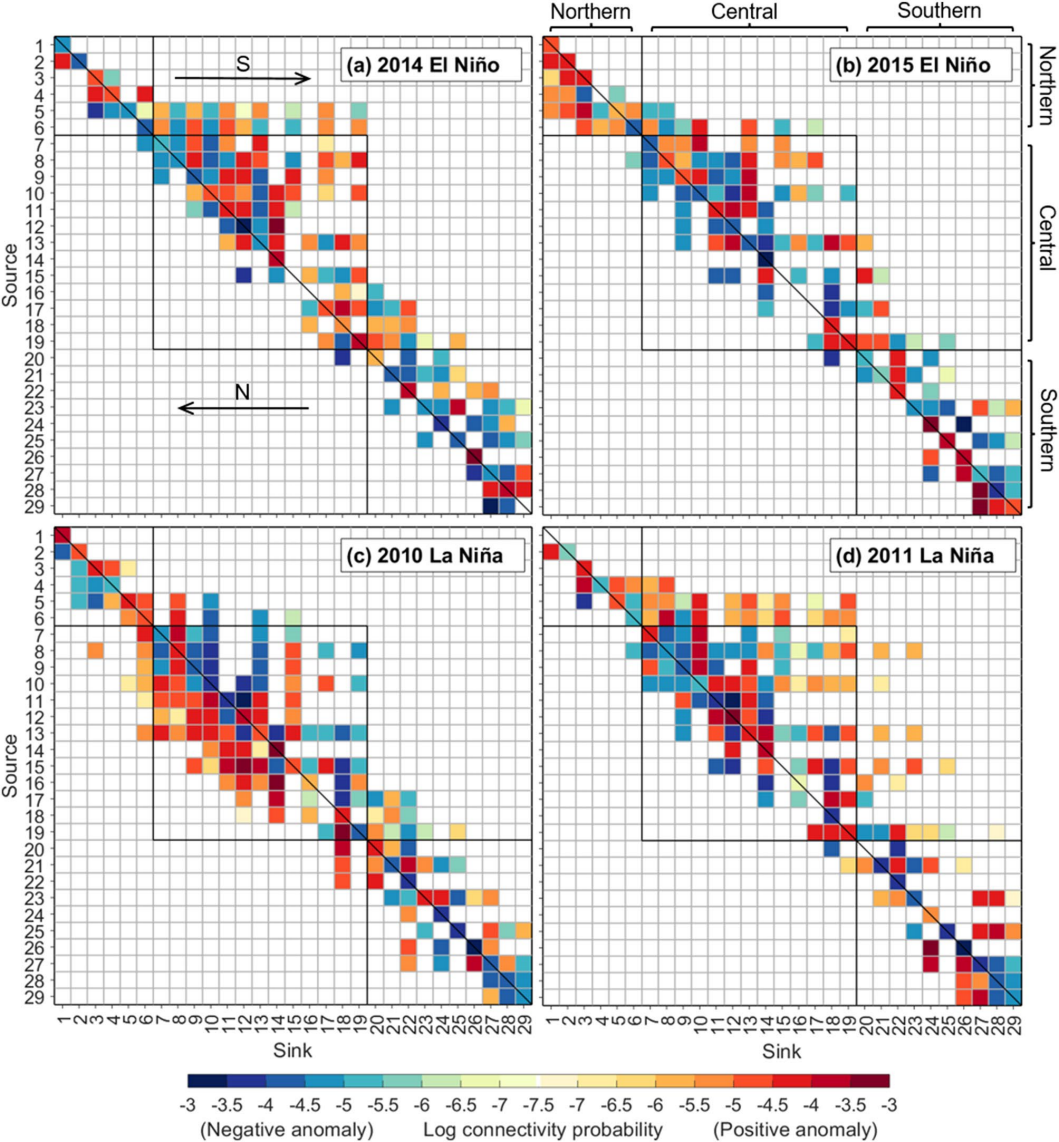
Connectivity matrices for modelled *L. carponotatus* larvae in the GBR, during (**a**) 2014 El Niño alert, (**b**) strong 2015 El Niño, (**c**) very strong 2010 La Niña, and (**d**) moderate 2011 La Niña. Positive and negative anomalies (referenced to the 2010–2017 mean connectivity) are represented by darker red–lighter orange and darker blue–lighter green shades (i.e. higher–lower values for both). Values along the diagonal represent larval retention, and values to the right or left of the diagonal indicate poleward (“S”) or equatorward (“N”) connectivity. Northern, central and southern GBR sectors are bordered by black lined squares. Regions are ordered from north to south following the same order as presented in the legend of the upper inset in Fig. 1. The figure was created using MATLAB v9.4, available at https://www.mathworks.com/products/matlab.html.

Poleward larval connectivity predominated throughout the central and southern GBR during the 2014 El Niño alert and strong 2015 El Niño events (79% and 71% of connections, respectively) (Fig. 4a,b). Some of the strongest poleward connectivity patterns throughout the central GBR over the study period occurred in 2014 (Fig. 4a) and resulted in central and southern regions largely receiving larvae from reefs to the north (columns 8–29; Fig. 4a). Stronger than average connectivity values were also observed in 2014 (e.g. columns 12, 14, 19; Fig. 4a). Similarly, higher poleward larval connectivity occurred towards particular central and southern regions in 2015 (e.g. columns 13, 16, 20; Fig. 4b). In 2014, poleward connectivity was strengthened from the southern half of the northern GBR towards the central GBR, including some of the longest connections (at least 600 km) over the study period (rows 5, 6; Fig. 4a). Conversely, in 2015, equatorward connectivity was strengthened throughout the northern GBR, resulting in the longest equatorward connections from northern reefs (at least ~ 300 km) over the study period (e.g. rows 4–6; Fig. 4b).

### Larval connectivity during the 2010 and 2011 La Niña events

During the very strong 2010 La Niña event, equatorward larval connectivity predominated throughout the central and (inner) southern GBR (51% of connections) (Fig. 4c). Larval dispersal patterns in 2010 indicated a reversal of directions compared to 2014 and 2015 El Niño events (Fig. 4a,b). Central GBR regions, as well as some northern and southern regions, were uniquely sourced by larvae from reefs to the south (e.g. columns 7, 11; Fig. 4c) and received higher than average larval supplies from these reefs (e.g. columns 6, 7–14, 22; Fig. 4c). The longest equatorward connections from central and southern GBR regions (at least ~ 250 km) over the study period occurred in 2010 (e.g. rows 8, 13, 27; Fig. 4c). In 2010, there were also poleward connections, although the majority of these exhibited below average connectivity values (Fig. 4c). In the moderate 2011 La Niña event, poleward larval connectivity predominated throughout the central and southern GBR (68% of connections), although equatorward connectivity was greater than during El Niño events (Fig. 4d). Also, poleward connectivity was strengthened from the southern half of the northern GBR towards the central GBR in 2011 (rows 4–6; Fig. 4d). The longest poleward connections (at least 600 km) over the study period occurred in 2011 (together with 2014) (e.g. rows 5, 6, 8; Fig. 4d).

## Discussion

The hydrodynamic conditions associated with ENSO events play an important role in regulating regional scale larval connectivity patterns in the GBR. Our findings show a highly connected system over the study period (2010–2017), whilst there were large interannual connectivity fluctuations associated with ENSO events. Our results indicate a reversal of the dominant flow in connectivity patterns in the central and southern GBR. Specifically, a transition occurred from a predominant poleward flow during weak SOI and El Niño conditions to a predominant equatorward flow during very strong SOI and extreme La Niña conditions. As ENSO is the most prominent source of year-to-year global climate variability, it is relevant to consider its impact on marine ecological processes, such as larval dispersal and connectivity patterns.

Temporal variability in larval dispersal patterns and recruitment have important implications for the replenishment of reef fish populations in the GBR^23,52^. Our results revealed temporal fluctuations in larval dispersal patterns in the GBR associated with different ENSO events and variations in the SOI. Similarly, research in tropical coral reef ecosystems showed that increases in coral reef fish larval supply can be strongly related to particular ENSO events^18^, and that strong year classes of coral reef fishes can correspond to the timing of ENSO events^53^. Furthermore, positive correlations have been found between the strength of the SOI and annual supply rates of new reef fish recruits^17^ and juvenile reef fish abundance^54^ at Ningaloo Reef in Western Australia. On the GBR, synchronous population fluctuations of coral reef fish have been correlated with ENSO^26^. These fish population increases often follow El Niño events, likely as a consequence of dispersal and environmental variation associated with ENSO^26^. In our study, we present the effect of the dispersal mechanism linked to ENSO on population connectivity patterns in the GBR, which may result in higher (or lower) rates of larval supply to reefs.

Large temporal fluctuations in recruitment dynamics can greatly influence fish population size^55^, recruitment to the fishery^56^, and larval subsidies expected from marine protected areas^57^. Our findings demonstrate that dispersal patterns in the GBR are highly variable in terms of magnitude and directionality over time. Furthermore, our results indicate that stronger, but also weaker and longer-distance connections, were associated with ENSO events. Importantly, graph theory (i.e. a framework for representation and analysis of larval connections) in the GBR showed that weaker edges (connections) are important for reef fish community structure across the system^25^. Accordingly, removing weaker edges resulted in a fragmented sparsely connected network^25^. Furthermore, coral reef fish larval connectivity patterns and large volatility in temporal dispersal have critical implications for the persistence and management of populations^20,58^, e.g. with stability arising from volatility^57^. Given the large variability exhibited over time in marine larval dispersal patterns, considering long-term data series is fundamental to better understand the temporal variation associated with large-scale climatic events.

Asymmetric (directional) connectivity has important consequences for conservation planning, as demonstrated across different habitats and organisms, including coral reef fish in the GBR^59^. Asymmetric connectivity may^60^ or may not^61^ have negative impacts on metapopulation persistence. Therefore, accounting for asymmetric dispersal when using metapopulation models is advised to better estimate persistence and manage populations^62^. Furthermore, asymmetric dispersal can influence genetic diversity throughout a species’ range^63^. Over the study period, our results demonstrate asymmetric larval connectivity in the central and southern GBR, with more prevalent poleward dispersal and less prevalent equatorward dispersal. We also showed that strong equatorward dispersal associated with extreme ENSO events, such as the very strong 2010 La Niña, would contribute to regional symmetry of dispersal (reversed flows). The importance of equatorward larval dispersal pulses on the GBR has been highlighted as a mechanism that maintains the integrity of reef fish metapopulations, also supporting the transfer of genetic information towards upstream reefs^23^. Implications of asymmetrical dispersal have also been extended to other GBR organisms, including corals^64^. Biophysical dispersal models and genetic surveys of broadcasting corals inferred asymmetric larval dispersal along the GBR, revealing more prevalent connections from north to south, with the strongest gene flow signals in the same direction^64^.

Previous research shows a strong association between westward Coral Sea transport and ENSO, increasing (or decreasing) following El Niño (or La Niña) events^8^. On the GBR, a surface layer along-shore current was described in the surroundings of Lizard Island in the southern part of the northern GBR, displaying interannual variability in the equatorward and poleward velocities (during austral spring, between 2008 and 2013)^47^. Adjacent to the southern half of the northern GBR, the westward flowing NVJ bifurcates in the Coral Sea, where the bifurcation latitude and current strength varied interannually under different ENSO events in our study (see Supplementary Results and Fig. S4). Importantly, these hydrodynamic changes were associated with temporal variation in bi-directional larval dispersal patterns around this region (~ 14°S–15°S). This temporal variation included, for example, the strongest equatorward connectivity patterns from northern GBR regions during the strong 2015 El Niño event, resulting from a southward shift in the bifurcation location. Furthermore, some of the strongest poleward connectivity patterns from the southern half of the northern GBR occurred during the 2011 La Niña event, associated with a northward shift in the bifurcation position.

Oceanic inflow from the Coral Sea on the central GBR circulation can influence the along- and across-shelf dispersal of spawn material from reefs^9^. In the present study, we found oceanic inflow from the EAC onto the central GBR shelf over the study period (see Supplementary Results and Fig. S4), with inflow enhancing variable poleward larval connectivity patterns, including cross-shelf connections from offshore reefs. However, during the very strong 2010 La Niña event, oceanic inflow was associated with cross-shelf and equatorward larval dispersal linking reefs. The 2010 La Niña event was associated with record rainfall in Queensland^28^, showing some of the greatest negative anomalies in sea surface salinity on the GBR (during austral spring–summer, between 2008 and 2015)^47^. Record rainfall in 2010 produced large river plumes along the GBR coast^16^, with predominantly equatorward along-shore flow, as suggested during strong southeasterly trades^15^. These circulation patterns were confirmed by our ocean, riverine discharge and wind circulation results during the 2010 La Niña event (see Supplementary Results and Figs. S4, S5, S6) and contributed to the strengthened equatorward larval dispersal.

The present modelling study supports the extensive connectivity displayed by fish genetics along the GBR^65,66^, including *L. carponotatus*^46^. Our results support the notion that genetic exchange does occur at contemporary (ecological) scales on the GBR. Also, our findings suggest that gene flow between most distant regions is favoured by larval exchange through intermediate reefs over time. Reef fish population connectivity (and associated low genetic differentiation) over regional scales has been proposed as a consequence of larval dispersal in a stepping-stone manner (e.g.^43^). We found large-scale multidirectional dispersal among GBR regions, as confirmed by realised larval dispersal patterns (genetic parentage analysis) of coral reef groupers in the southern GBR^24^. Furthermore, we highlight the relevance of ENSO-linked dispersal events to enhance multidirectional connectivity throughout the GBR, which would contribute larvae for the replenishment of regional reefs.

The findings presented here reveal the effects that ENSO can have on the interannual marine connectivity patterns in the world’s largest coral reef ecosystem. The frequency of extreme El Niño and La Niña events is projected to double under unabated climate change^3,4^, and ENSO rainfall responses are expected to intensify^2,67^. Therefore, these changes may have important connectivity implications not only for the GBR but also for other regions globally that are affected by ENSO. With an increase in the frequency of extreme climatic events, the stability, strength and distance of larval connections may be affected in the GBR. Additionally, the asymmetry of connectivity patterns and mixing of genetic traits may also be affected. In fact, these factors could potentially influence the metapopulation persistence, community structure, or adaptive potential of reef species in the region. Observations of the ocean–atmosphere system have shown that no two ENSO events are the same^2^. Therefore, ENSO diversity, together with the associated connectivity impacts, should be considered when evaluating marine connectivity patterns.

## Conclusion

This study demonstrates the influence of ENSO on GBR larval connectivity using interannual biophysical modelling. A well-connected GBR system is maintained over time, given the interannual variability of connectivity among regions. Bi-directional larval connectivity is exhibited among regions, with changes in dispersal patterns associated with different ENSO events. A predominant, but variable poleward connectivity is exhibited throughout the central and southern GBR during different ENSO phases and strengths. However, extreme ENSO events, such as the very strong 2010 La Niña, can promote stronger equatorward larval dispersal from central and southern regions. In the far northern GBR, larval connectivity is predominantly equatorward. However, supply from reefs in the southern half of the northern GBR to reefs further north (or south) increases with ENSO events. Interannual modelled larval connectivity highlights the role of ENSO on connectivity patterns and the need to understand the stability of connections among reefs over time.

## Supplementary Information

Supplementary Information.

## Data Availability

The eReefs model simulations were produced as part of the eReefs project (eReefs.info), a collaboration between the Science Industry Endowment Fund (SIEF), the Commonwealth Scientific Industrial Research Organisation (CSIRO), the Australian Institute of Marine Science (AIMS), the Bureau of Meteorology (BOM), and the Great Barrier Reef Foundation (GBRF), with support from BHP Billiton Mitsubishi Alliance, the Australian and Queensland governments, and with observations obtained through the Integrated Marine Observing System (IMOS). The ACCESS-R data are available through the Bureau of Meteorology (http://www.bom.gov.au/). The eReefs model current, wind and salinity data are available through the eReefs Catalog (https://thredds.ereefs.aims.gov.au/thredds/catalog.html; https://thredds.ereefs.aims.gov.au/thredds/s3catalogue/aims-ereefs-public-prod/derived/ncaggregate/ereefs/gbr4_v2/catalog.html). The daily eReefs model data has been gridded onto a uniform grid from the original dataset. Original data—eReefs v2 model data—are available on the National Computational Infrastructure (NCI) Thredds server at https://dapds00.nci.org.au/thredds/catalog/fx3/gbr4_v2/catalog.html. AIMS eReefs server and derived data products are described at https://ereefs.aims.gov.au/ereefs-aims/help/how-to-manually-download-aims-ereefs-data. The shapefile containing the “Complete GBR Reef and Island Features”^68^ is available through the eAtlas server © Great Barrier Reef Marine Park Authority 2014 (https://eatlas.org.au/data/uuid/d2396b2c-68d4-4f4b-aab0-52f7bc4a81f5). Updated data available at http://www.gbrmpa.gov.au/geoportal/. The CSIRO Connectivity Interface (Connie3) is available at https://connie.csiro.au/.
