# Supplementary material for: The El Niño Southern Oscillation drives multidirectional inter-reef larval connectivity in the Great Barrier Reef
Source: Sci Rep. 2022 Dec 9;12:21290. doi: 10.1038/s41598-022-25629-w (PMC9734173; doi:10.1038/s41598-022-25629-w)
Supplement: Supplementary file 1 — Supplementary Information. [file 41598_2022_25629_MOESM1_ESM.pdf]

Supplementary information

The El Niño Southern Oscillation drives multidirectional inter-reef larval connectivity in the Great Barrier Reef

Rodrigo Gurdek-Bas[1,2,3*], Jessica A. Benthuysen[3], Hugo B. Harrison[3,4], Kyall R. Zenger[2] & Lynne van Herwerden[2]

[1]AIMS@JCU, Division of Research & Innovation, James Cook University and Australian Institute of Marine Science, Townsville, 4811, Australia. [2]College of Science and Engineering, James Cook University, Townsville, 4811, Australia. [3]Australian Institute of Marine Science, Townsville, 4810, Australia. [4]ARC Centre of Excellence for Coral Reef Studies, James Cook University, Townsville, 4811, Australia.

email: rodrigo.gurdek@my.jcu.edu.au

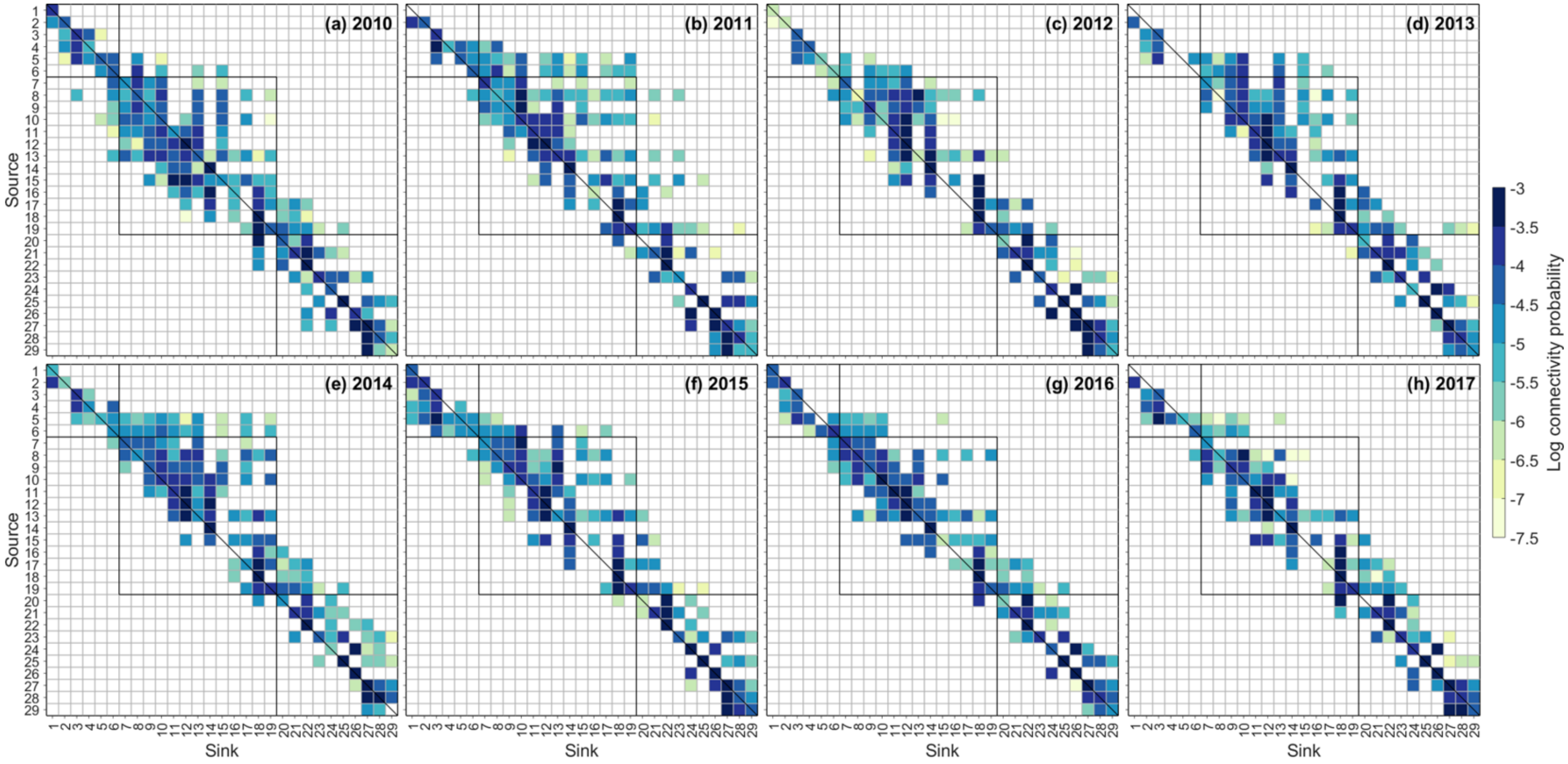

Figure S1. Connectivity matrices for modelled *L. carponotatus* larvae in the GBR, during (**a**) very strong 2010 La Niña, (**b**) moderate 2011 La Niña, (**c**) 2012 neutral, (**d**) 2013 neutral, (**e**) 2014 El Niño alert, (**f**) strong 2015 El Niño, (**g**) 2016 neutral–La Niña, and (**h**) 2017 neutral–La Niña. Values along the diagonal represent larval retention. Values to the right or left of the diagonal indicate poleward or equatorward connectivity, respectively. Northern, central and southern GBR sectors are bordered by black lined squares. Regions are ordered from north to south following the same order as stated in the legend of the upper inset, Fig. 1. The figure was created using MATLAB v9.4, available at https://www.mathworks.com/products/matlab.html.

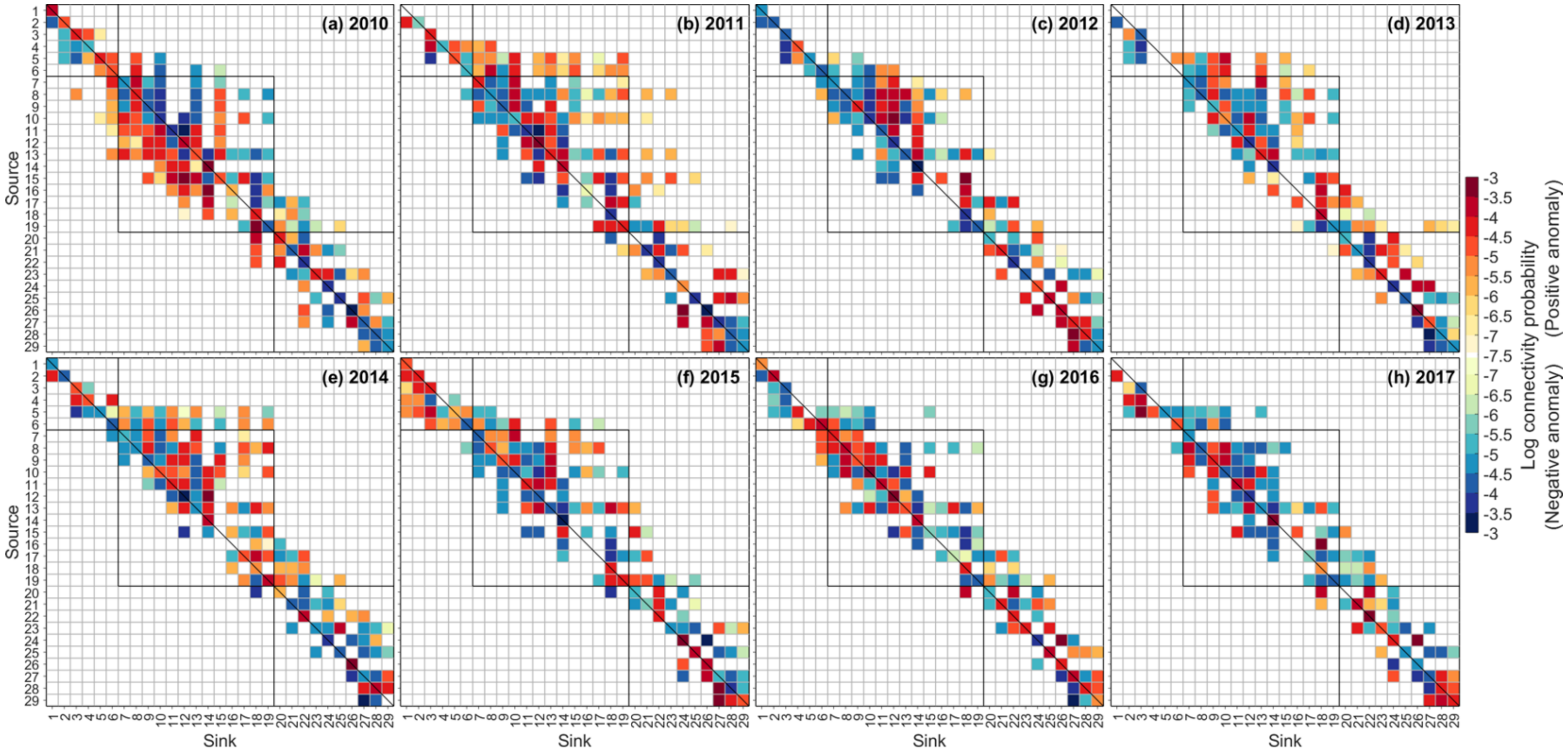

Figure S2. Connectivity matrices for modelled *L. carponotatus* larvae in the GBR, during (**a**) very strong 2010 La Niña, (**b**) moderate 2011 La Niña, (**c**) 2012 neutral, (**d**) 2013 neutral, (**e**) 2014 El Niño alert, (**f**) strong 2015 El Niño, (**g**) 2016 neutral–La Niña, and (**h**) 2017 neutral–La Niña. Positive and negative anomalies are referenced to the 2010–2017 mean connectivity. Values along the diagonal represent larval retention. Values to the right or left of the diagonal indicate poleward or equatorward connectivity, respectively. Northern, central and southern GBR sectors are bordered by black lined squares. Regions are ordered from north to south following the same order as stated in the legend of the upper inset, Fig. 1. The figure was created using MATLAB v9.4, available at https://www.mathworks.com/products/matlab.html.

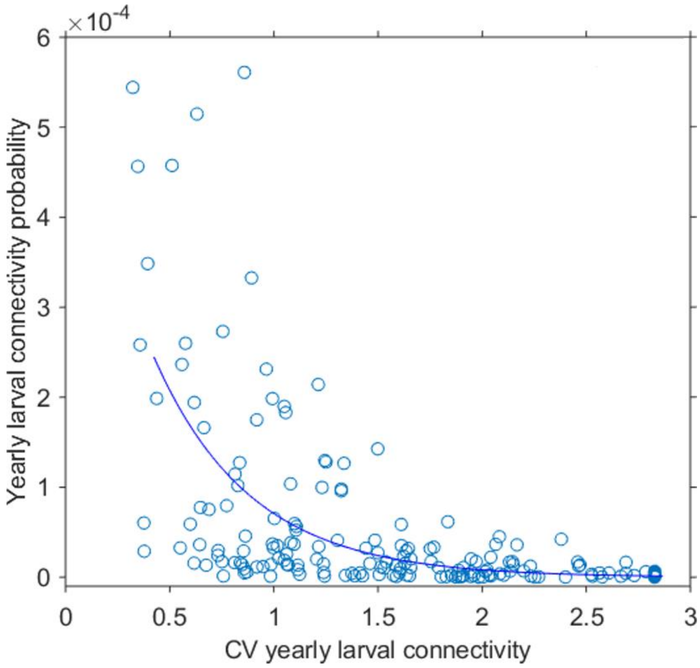

Figure S3. Relationship between inter-regional larval connectivity probabilities (averaged over eight *L. carponotatus* main spawning seasons from 2010 to 2017) and the coefficient of variation (CV) of those connections in the GBR. Connectivity probability and CV values correspond to those of Figs. 2a and 2b. The particle release locations are shown in Fig. 1.

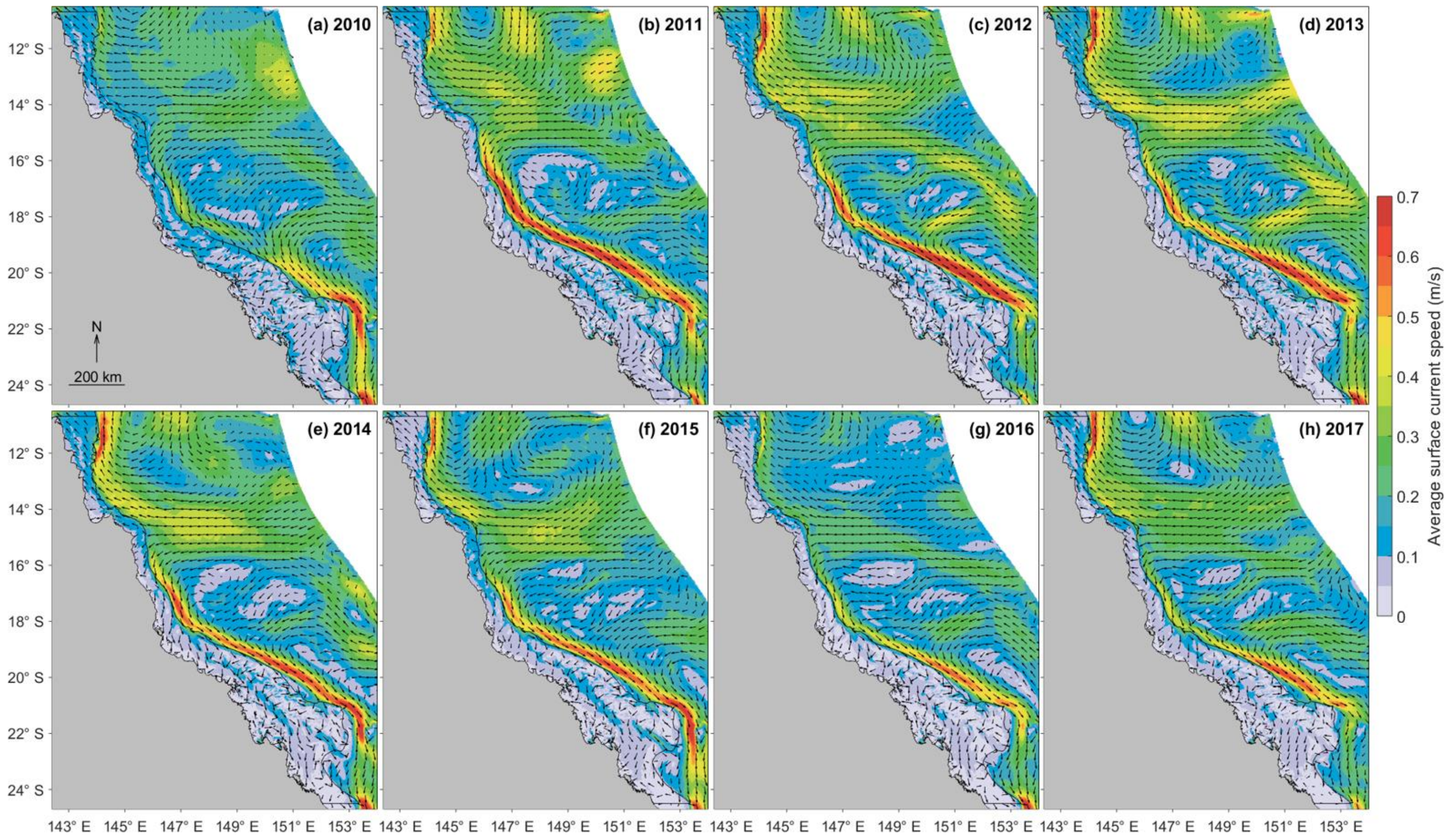

Figure S4. Average surface current velocity (m s^{-1}) (October–January) over the GBR and Coral Sea for (**a**) very strong 2010 La Niña, (**b**) moderate 2011 La Niña, (**c**) 2012 neutral, (**d**) 2013 neutral, (**e**) 2014 El Niño alert, (**f**) strong 2015 El Niño, (**g**) 2016 neutral–La Niña, and (**h**) 2017 neutral–La Niña. Current directions are indicated by arrows, and speeds are shaded. The black line offshore (following the length of the coast) delimits the GBR shelf and corresponds to the 100 m isobath. The figure was created using MATLAB v9.4, available at https://www.mathworks.com/products/matlab.html.

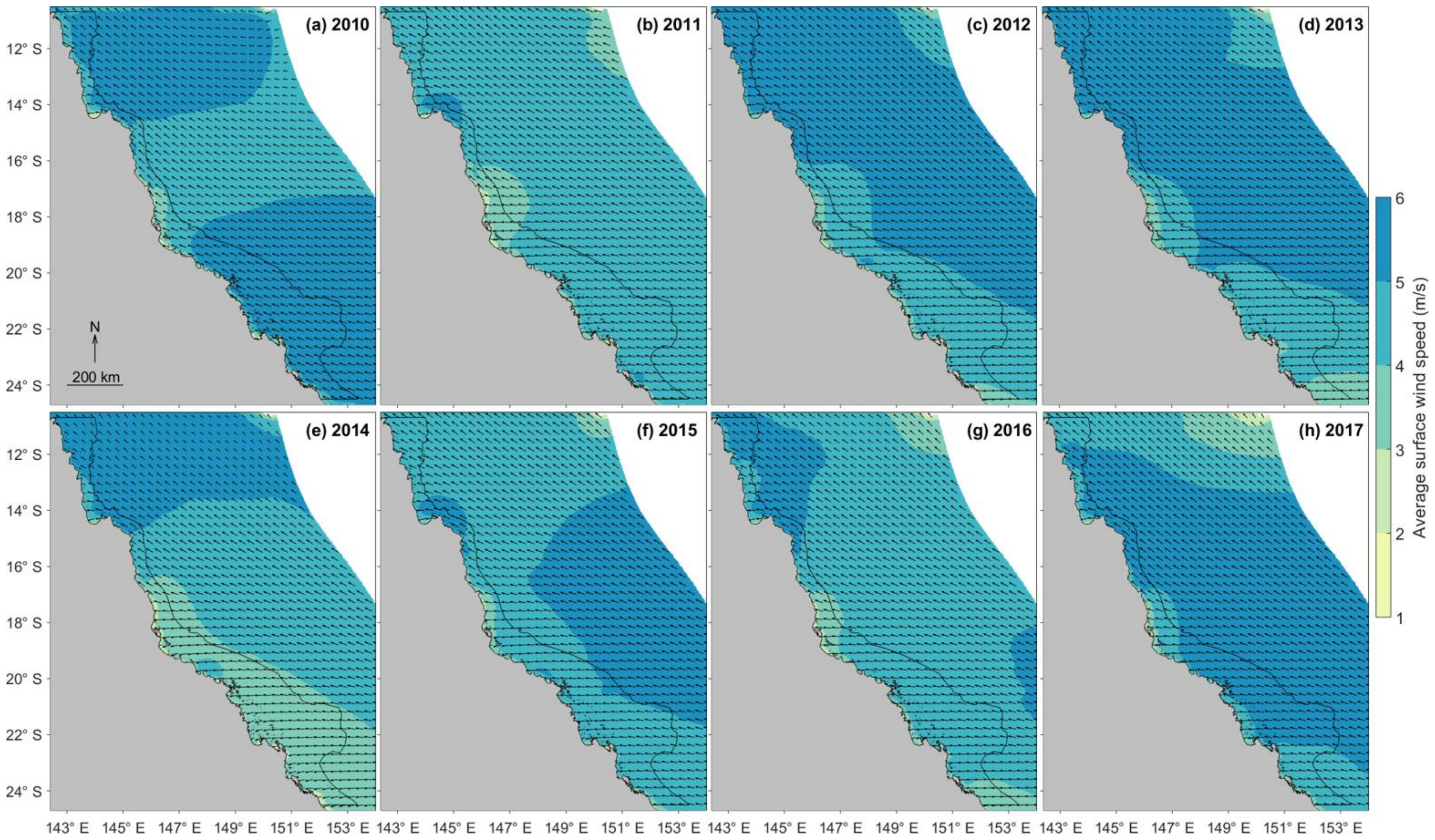

Figure S5. Average surface wind velocity (m s^{-1}) (October–January) over the GBR and Coral Sea for (**a**) very strong 2010 La Niña, (**b**) moderate 2011 La Niña, (**c**) 2012 neutral, (**d**) 2013 neutral, (**e**) 2014 El Niño alert, (**f**) strong 2015 El Niño, (**g**) 2016 neutral–La Niña, and (**h**) 2017 neutral–La Niña. Current directions are indicated by arrows, and speeds are shaded. The black line offshore (following the length of the coast) delimits the GBR shelf and corresponds to the 100 m isobath. The figure was created using MATLAB v9.4, available at https://www.mathworks.com/products/matlab.html.

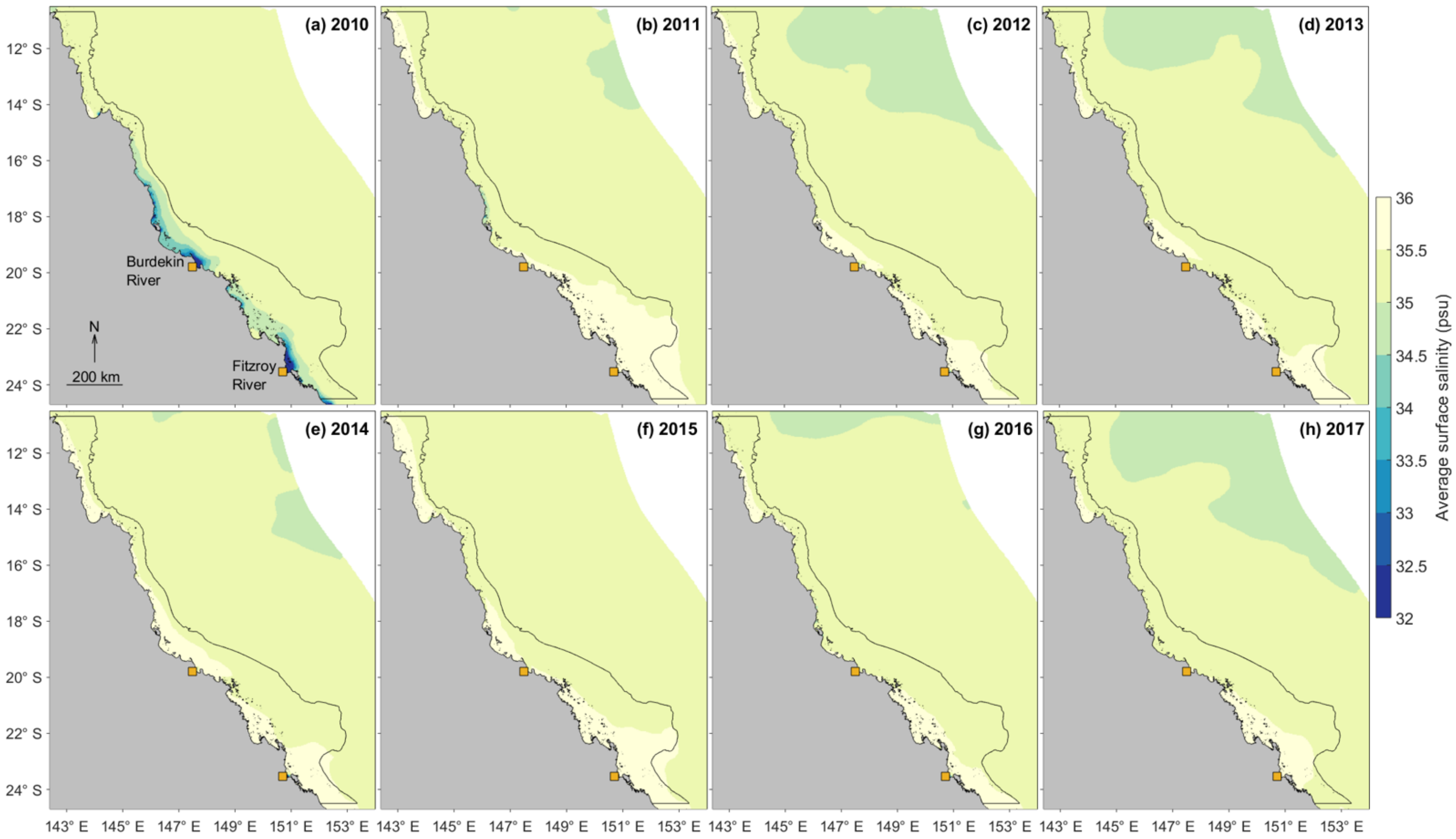

Figure S6. Average surface salinity (psu) (October–January) over the GBR and Coral Sea for (**a**) very strong 2010 La Niña, (**b**) moderate 2011 La Niña, (**c**) 2012 neutral, (**d**) 2013 neutral, (**e**) 2014 El Niño alert, (**f**) strong 2015 El Niño, (**g**) 2016 neutral–La Niña, and (**h**) 2017 neutral–La Niña. The black line offshore (following the length of the coast) delimits the GBR shelf and corresponds to the 100 m isobath. The figure was created using MATLAB v9.4, available at https://www.mathworks.com/products/matlab.html.

Supplementary Results

Ocean, riverine discharge and wind circulation patterns. Ocean and wind circulation patterns were investigated in the Great Barrier Reef (GBR) and Coral Sea for the larval dispersal periods from 2010 to 2017. The location of the North Vanuatu Jet (NVJ) bifurcation, into the equatorward Gulf of Papua Current (GPC) and poleward East Australian Current (EAC), varied between years. The NVJ bifurcation location showed a gradual shift over time from the northernmost position (at ~14.3°S) during the very strong 2010 La Niña event to the southernmost position (at ~15°S) during the strong 2015 El Niño event (see Supplementary Fig. S4). In the far northern GBR, an equatorward transport (adjacent to the GPC) occurred over the study period, while GBR circulation around the NVJ bifurcation location (southern half of the northern GBR) generally varied between poleward and equatorward directions according to the bifurcation position (see Supplementary Fig. S4). The EAC was associated with oceanic inflow onto the GBR shelf at ~16.5°S and major oceanic inflow at ~18°S (central GBR), resulting in a predominant poleward flow over the central and southern mid-shelf over the study period, except during the very strong 2010 La Niña event (see Supplementary Fig. S4). The dominant wind direction in the GBR and Coral Sea was from the southeast (southeasterly winds) (see Supplementary Fig. S5).

The 2014 and 2015 El Niño events. A strong NVJ bifurcation into the GPC and EAC occurred during the 2014 El Niño alert and the strong 2015 El Niño events, particularly in 2014 (see Supplementary Fig. S4). During El Niño events, an EAC surface speed of ~0.6 m s^{-1} occurred along the outside of the central and southern GBR (see Supplementary Fig. S4). The poleward flowing EAC was associated with a predominant poleward flow (up to ~0.15 m s^{-1}) over the central and southern GBR shelf during El Niño conditions, though this poleward flow was strengthened in 2014 (see Supplementary Fig. S4). The 2014 ocean circulation patterns were associated with weak easterly–southeasterly winds of ~3–4 m s^{-1} over the central and southern GBR (see Supplementary Fig. S5). In 2015, easterly–southeasterly wind speeds increased to ~4–5 m s^{-1} over the central and southern sectors (see Supplementary Fig. S5).

The 2010 and 2011 La Niña events. The NVJ and EAC were weakest during the very strong 2010 La Niña event (see Supplementary Fig. S4). This circulation pattern was

associated with the strongest southeasterly winds (~5–6 m s^{-1}) over the central and southern GBR (see Supplementary Fig. S5). In 2010, oceanic inflow onto the central GBR resulted in an across-shelf and equatorward transport (see Supplementary Fig. S4). Over the study period, river discharge to the GBR was very low, except in 2010 (see Supplementary Fig. S6). In 2010, high river discharges occurred, including those from major rivers, such as the Burdekin (19°39’S, 147°30’E) and Fitzroy (23°31’S, 150°53’E) Rivers (see Supplementary Fig. S6). Consequently, low-salinity along-shore equatorward plumes occurred along the inner- and mid-shelves in the northern, central and southern GBR (notably from December) (see Supplementary Fig. S6). The effect of the Coral Sea circulation, winds and river flows in 2010 resulted in a predominantly equatorward circulation over much of the GBR (see Supplementary Fig. S4). During the moderate 2011 La Niña event, a strong EAC developed adjacent to the central and southern GBR, and this circulation was associated with a predominant poleward transport in the GBR (see Supplementary Fig. S4). In 2011, southeasterly winds of ~4–5 m s^{-1} occurred over the central and southern GBR (see Supplementary Fig. S5).
